# Supplementary material for: Patterns of postnatal weight gain and its predictors among preterm very low birth weight neonates born in Bahir-Dar city public hospitals, 2022: A cross sectional study
Source: PLoS One. 2025 Feb 12;20(2):e0315573. doi: 10.1371/journal.pone.0315573 (PMC11819586; doi:10.1371/journal.pone.0315573)
Supplement: S1 File — (DOCX) [file pone.0315573.s001.docx]

Smoothed percentiles tables of preterm VLBW neonates with respect to their gestational age (29-36 weeks). Supplementary tables (S1-S8)

Table S1: Smoothed percentiles for 29 weeks gestation (N=48)

| N | days | 3^rd^ | 10^th^ | 50^th^ | 90^th^ | 97^th^ | mean | SD |
| --- | --- | --- | --- | --- | --- | --- | --- | --- |
| 48 | 1 | 993 | 1008 | 1192 | 1397 | 1434 | 1213 | 135 |
| 48 | 2 | 971 | 988 | 1172 | 1372 | 1416 | 1180 | 138 |
| 48 | 3 | 949 | 968 | 1153 | 1348 | 1400 | 1162 | 142 |
| 48 | 4 | 930 | 951 | 1136 | 1325 | 1385 | 1143 | 140 |
| 48 | 5 | 914 | 935 | 1121 | 1304 | 1371 | 1127 | 134 |
| 48 | 6 | 901 | 923 | 1111 | 1288 | 1361 | 1117 | 137 |
| 48 | 7 | 892 | 915 | 1106 | 1277 | 1352 | 1109 | 138 |
| 48 | 8 | 888 | 911 | 1107 | 1272 | 1344 | 1102 | 139 |
| 48 | 9 | 889 | 912 | 1113 | 1272 | 1336 | 1112 | 132 |
| 48 | 10 | 893 | 916 | 1125 | 1277 | 1329 | 1115 | 147 |
| 48 | 11 | 899 | 923 | 1140 | 1284 | 1324 | 1121 | 140 |
| 48 | 12 | 909 | 932 | 1160 | 1291 | 1322 | 1138 | 134 |
| 48 | 13 | 920 | 942 | 1181 | 1298 | 1323 | 1148 | 133 |
| 48 | 14 | 931 | 953 | 1202 | 1304 | 1326 | 1164 | 131 |
| 48 | 15 | 943 | 964 | 1220 | 1309 | 1330 | 1171 | 132 |
| 45 | 16 | 954 | 975 | 1235 | 1315 | 1335 | 1181 | 124 |
| 44 | 17 | 965 | 985 | 1247 | 1322 | 1342 | 1177 | 131 |
| 42 | 18 | 974 | 995 | 1260 | 1332 | 1352 | 1101 | 351 |
| 41 | 19 | 983 | 1004 | 1274 | 1345 | 1365 | 1202 | 136 |
| 38 | 20 | 991 | 1012 | 1289 | 1360 | 1380 | 1236 | 132 |
| 38 | 21 | 998 | 1019 | 1304 | 1377 | 1398 | 1251 | 133 |
| 31 | 22 | 1005 | 1026 | 1320 | 1395 | 1418 | 1248 | 144 |
| 30 | 23 | 1012 | 1033 | 1338 | 1414 | 1440 | 1272 | 150 |
| 30 | 24 | 1020 | 1040 | 1355 | 1434 | 1461 | 1295 | 154 |
| 30 | 25 | 1028 | 1047 | 1372 | 1453 | 1481 | 1306 | 150 |
| 29 | 26 | 1035 | 1054 | 1386 | 1471 | 1499 | 1324 | 153 |
| 29 | 27 | 1041 | 1061 | 1399 | 1488 | 1515 | 1337 | 156 |
| 29 | 28 | 1048 | 1068 | 1409 | 1502 | 1528 | 1353 | 159 |
| 28 | 29 | 1056 | 1075 | 1419 | 1515 | 1541 | 1352 | 155 |
| 28 | 30 | 1065 | 1082 | 1428 | 1527 | 1553 | 1366 | 162 |
| 28 | 31 | 1075 | 1091 | 1436 | 1539 | 1564 | 1380 | 172 |
| 24 | 32 | 1086 | 1100 | 1444 | 1551 | 1576 | 1370 | 170 |
| 22 | 33 | 1097 | 1110 | 1453 | 1563 | 1587 | 1382 | 179 |

Table S2: Smoothed percentiles for 30 weeks gestation (N=31)

| N | days | 3^rd^ | 10^th^ | 50^th^ | 90^th^ | 97^th^ | mean | SD |
| --- | --- | --- | --- | --- | --- | --- | --- | --- |
| 31 | 1 | 924 | 1009 | 1274 | 1316 | 1335 | 1234 | 129 |
| 31 | 2 | 915 | 995 | 1261 | 1302 | 1319 | 1202 | 130 |
| 31 | 3 | 912 | 985 | 1249 | 1295 | 1311 | 1183 | 141 |
| 31 | 4 | 912 | 977 | 1240 | 1294 | 1312 | 1153 | 123 |
| 31 | 5 | 915 | 972 | 1234 | 1298 | 1319 | 1155 | 127 |
| 31 | 6 | 922 | 970 | 1231 | 1307 | 1330 | 1153 | 121 |
| 31 | 7 | 931 | 971 | 1231 | 1317 | 1344 | 1153 | 121 |
| 31 | 8 | 943 | 975 | 1233 | 1330 | 1358 | 1170 | 132 |
| 31 | 9 | 956 | 982 | 1237 | 1344 | 1373 | 1189 | 139 |
| 31 | 10 | 972 | 991 | 1243 | 1360 | 1390 | 1208 | 150 |
| 31 | 11 | 989 | 1004 | 1251 | 1375 | 1405 | 1231 | 143 |
| 28 | 12 | 1007 | 1019 | 1260 | 1388 | 1417 | 1245 | 136 |
| 28 | 13 | 1026 | 1037 | 1269 | 1399 | 1425 | 1256 | 144 |
| 28 | 14 | 1047 | 1056 | 1277 | 1407 | 1430 | 1265 | 138 |
| 28 | 15 | 1068 | 1077 | 1285 | 1412 | 1433 | 1282 | 132 |
| 28 | 16 | 1088 | 1097 | 1293 | 1417 | 1435 | 1291 | 122 |
| 24 | 17 | 1107 | 1117 | 1301 | 1420 | 1436 | 1292 | 122 |
| 20 | 18 | 1127 | 1137 | 1308 | 1424 | 1439 | 1280 | 96 |
| 20 | 19 | 1147 | 1157 | 1316 | 1427 | 1442 | 1292 | 102 |
| 20 | 20 | 1167 | 1176 | 1324 | 1431 | 1448 | 1306 | 104 |
| 16 | 21 | 1184 | 1194 | 1333 | 1437 | 1458 | 1324 | 116 |
| 16 | 22 | 1199 | 1210 | 1344 | 1448 | 1475 | 1338 | 123 |

Table S3: Smoothed percentiles for 31 weeks gestation (N=39)

| N | Days | 3^rd^ | 10^th^ | 50^th^ | 90^th^ | 97^th^ | mean | SD |
| --- | --- | --- | --- | --- | --- | --- | --- | --- |
| 39 | 1 | 1158 | 1194 | 1345 | 1397 | 1400 | 1317 | 92 |
| 39 | 2 | 1133 | 1167 | 1328 | 1376 | 1380 | 1293 | 91 |
| 39 | 3 | 1110 | 1142 | 1313 | 1357 | 1363 | 1271 | 92 |
| 39 | 4 | 1089 | 1123 | 1299 | 1348 | 1356 | 1249 | 93 |
| 39 | 5 | 1070 | 1112 | 1287 | 1348 | 1358 | 1242 | 105 |
| 39 | 6 | 1052 | 1107 | 1269 | 1354 | 1366 | 1234 | 109 |
| 39 | 7 | 1036 | 1099 | 1248 | 1364 | 1377 | 1231 | 117 |
| 39 | 8 | 1026 | 1085 | 1228 | 1378 | 1389 | 1230 | 127 |
| 39 | 9 | 1025 | 1070 | 1215 | 1394 | 1402 | 1227 | 135 |
| 39 | 10 | 1038 | 1065 | 1215 | 1409 | 1417 | 1235 | 145 |
| 39 | 11 | 1062 | 1077 | 1229 | 1424 | 1435 | 1250 | 143 |
| 39 | 12 | 1091 | 1100 | 1253 | 1438 | 1453 | 1267 | 144 |
| 39 | 13 | 1120 | 1129 | 1278 | 1453 | 1469 | 1286 | 132 |
| 39 | 14 | 1145 | 1158 | 1302 | 1470 | 1484 | 1308 | 121 |
| 39 | 15 | 1167 | 1186 | 1321 | 1491 | 1502 | 1325 | 118 |
| 38 | 16 | 1188 | 1215 | 1336 | 1509 | 1521 | 1344 | 116 |
| 38 | 17 | 1207 | 1241 | 1345 | 1518 | 1541 | 1368 | 115 |
| 34 | 18 | 1221 | 1259 | 1349 | 1522 | 1563 | 1361 | 106 |
| 34 | 19 | 1229 | 1267 | 1355 | 1530 | 1586 | 1377 | 109 |
| 34 | 20 | 1233 | 1267 | 1365 | 1540 | 1609 | 1390 | 115 |

Table S4: Smoothed percentiles for 32 weeks gestation (N=40)

| N | Days | 3^rd^ | 10^th^ | 50^th^ | 90^th^ | 97^th^ | mean | SD |
| --- | --- | --- | --- | --- | --- | --- | --- | --- |
| 40 | 1 | 1157 | 1192 | 1344 | 1395 | 1398 | 1317 | 92 |
| 40 | 2 | 1133 | 1167 | 1329 | 1375 | 1379 | 1293 | 91 |
| 40 | 3 | 1110 | 1143 | 1313 | 1359 | 1365 | 1271 | 92 |
| 40 | 4 | 1089 | 1125 | 1299 | 1350 | 1358 | 1249 | 93 |
| 40 | 5 | 1069 | 1113 | 1285 | 1349 | 1358 | 1242 | 105 |
| 40 | 6 | 1051 | 1105 | 1268 | 1354 | 1365 | 1234 | 109 |
| 40 | 7 | 1036 | 1096 | 1248 | 1364 | 1376 | 1231 | 117 |
| 40 | 8 | 1027 | 1083 | 1229 | 1378 | 1388 | 1230 | 127 |
| 40 | 9 | 1027 | 1071 | 1217 | 1393 | 1402 | 1227 | 135 |
| 40 | 10 | 1040 | 1068 | 1218 | 1408 | 1417 | 1235 | 145 |
| 40 | 11 | 1063 | 1079 | 1231 | 1423 | 1435 | 1250 | 143 |
| 40 | 12 | 1090 | 1101 | 1252 | 1438 | 1452 | 1267 | 144 |
| 40 | 13 | 1119 | 1129 | 1277 | 1454 | 1468 | 1286 | 132 |
| 40 | 14 | 1144 | 1158 | 1300 | 1471 | 1484 | 1308 | 121 |
| 40 | 15 | 1167 | 1186 | 1319 | 1490 | 1502 | 1325 | 118 |
| 38 | 16 | 1188 | 1214 | 1334 | 1506 | 1521 | 1344 | 116 |
| 38 | 17 | 1206 | 1238 | 1343 | 1516 | 1542 | 1368 | 115 |
| 34 | 18 | 1219 | 1256 | 1349 | 1523 | 1564 | 1361 | 106 |
| 35 | 19 | 1229 | 1267 | 1358 | 1532 | 1587 | 1377 | 109 |
| 35 | 20 | 1237 | 1274 | 1374 | 1546 | 1608 | 1390 | 115 |
| 34 | 21 | 1246 | 1285 | 1395 | 1565 | 1628 | 1407 | 118 |
| 34 | 22 | 1256 | 1301 | 1416 | 1585 | 1645 | 1433 | 116 |
| 34 | 23 | 1265 | 1318 | 1436 | 1606 | 1661 | 1438 | 119 |

Table S5: Smoothed percentiles for 33 weeks of gestation (N=62)

| N | days | 3^rd^ | 10^th^ | 50^th^ | 90^th^ | 97^th^ | mean | SD |
| --- | --- | --- | --- | --- | --- | --- | --- | --- |
| 62 | 1 | 988 | 1054 | 1379 | 1440 | 1456 | 1327 | 145 |
| 62 | 2 | 964 | 996 | 1331 | 1431 | 1445 | 1289 | 148 |
| 62 | 3 | 940 | 957 | 1291 | 1415 | 1432 | 1267 | 153 |
| 62 | 4 | 920 | 943 | 1264 | 1401 | 1425 | 1245 | 159 |
| 62 | 5 | 904 | 951 | 1252 | 1391 | 1450 | 1239 | 159 |
| 62 | 6 | 893 | 972 | 1256 | 1390 | 1475 | 1245 | 157 |
| 62 | 7 | 885 | 998 | 1273 | 1398 | 1500 | 1250 | 162 |
| 62 | 8 | 881 | 1023 | 1293 | 1412 | 1535 | 1257 | 166 |
| 62 | 9 | 887 | 1044 | 1309 | 1430 | 1563 | 1265 | 170 |
| 62 | 10 | 907 | 1064 | 1319 | 1449 | 1577 | 1286 | 168 |
| 62 | 11 | 934 | 1080 | 1329 | 1470 | 1591 | 1299 | 168 |
| 62 | 12 | 937 | 1091 | 1343 | 1492 | 1606 | 1313 | 169 |
| 62 | 13 | 941 | 1096 | 1360 | 1514 | 1622 | 1327 | 173 |
| 62 | 14 | 942 | 1100 | 1375 | 1536 | 1636 | 1341 | 178 |
| 62 | 15 | 949 | 1109 | 1386 | 1552 | 1637 | 1348 | 186 |
| 57 | 16 | 954 | 1126 | 1390 | 1557 | 1639 | 1358 | 188 |
| 54 | 17 | 955 | 1144 | 1391 | 1548 | 1644 | 1366 | 193 |
| 48 | 18 | 961 | 1161 | 1395 | 1529 | 1650 | 1332 | 173 |
| 48 | 19 | 961 | 1178 | 1401 | 1520 | 1650 | 1350 | 172 |
| 47 | 20 | 975 | 1198 | 1411 | 1524 | 1656 | 1368 | 169 |
| 45 | 21 | 989 | 1218 | 1423 | 1541 | 1658 | 1379 | 170 |
| 44 | 22 | 1001 | 1239 | 1437 | 1564 | 1676 | 1390 | 176 |

Table S6: Smoothed percentiles for 34 week gestation (N=55)

| N | days | 3^rd^ | 10^th^ | 50^th^ | 90^th^ | 97^th^ | mean | SD |
| --- | --- | --- | --- | --- | --- | --- | --- | --- |
| 55 | 1 | 1202 | 1256 | 1398 | 1474 | 1485 | 1381 | 89 |
| 55 | 2 | 1171 | 1217 | 1370 | 1454 | 1462 | 1352 | 85 |
| 55 | 3 | 1140 | 1178 | 1343 | 1438 | 1446 | 1325 | 96 |
| 55 | 4 | 1115 | 1153 | 1321 | 1427 | 1440 | 1306 | 99 |
| 55 | 5 | 1105 | 1145 | 1305 | 1424 | 1444 | 1296 | 104 |
| 55 | 6 | 1107 | 1150 | 1298 | 1428 | 1459 | 1305 | 100 |
| 55 | 7 | 1109 | 1152 | 1300 | 1438 | 1480 | 1304 | 115 |
| 55 | 8 | 1106 | 1154 | 1310 | 1449 | 1501 | 1305 | 123 |
| 55 | 9 | 1101 | 1161 | 1325 | 1460 | 1517 | 1314 | 125 |
| 55 | 10 | 1102 | 1172 | 1340 | 1472 | 1531 | 1321 | 132 |
| 55 | 11 | 1112 | 1185 | 1348 | 1483 | 1542 | 1330 | 130 |
| 52 | 12 | 1129 | 1203 | 1352 | 1495 | 1551 | 1345 | 129 |
| 52 | 13 | 1151 | 1225 | 1359 | 1509 | 1555 | 1364 | 127 |
| 48 | 14 | 1175 | 1247 | 1370 | 1526 | 1557 | 1370 | 115 |
| 48 | 15 | 1199 | 1267 | 1382 | 1545 | 1567 | 1393 | 117 |
| 47 | 16 | 1220 | 1286 | 1393 | 1564 | 1581 | 1410 | 113 |
| 46 | 17 | 1237 | 1301 | 1406 | 1581 | 1594 | 1419 | 111 |
| 46 | 18 | 1253 | 1316 | 1418 | 1596 | 1608 | 1437 | 110 |
| 46 | 19 | 1269 | 1329 | 1427 | 1607 | 1625 | 1456 | 112 |
| 40 | 20 | 1285 | 1341 | 1433 | 1612 | 1642 | 1448 | 98 |

Table S7: Smoothed percentiles for 35 week gestation (N=67)

| N | days | 3^rd^ | 10^th^ | 50^th^ | 90^th^ | 97^th^ | mean | SD |
| --- | --- | --- | --- | --- | --- | --- | --- | --- |
| 67 | 1 | 889 | 1130 | 1411 | 1484 | 1492 | 1359 | 162 |
| 67 | 2 | 874 | 1096 | 1390 | 1450 | 1471 | 1328 | 158 |
| 67 | 3 | 859 | 1067 | 1369 | 1424 | 1454 | 1306 | 155 |
| 67 | 4 | 842 | 1045 | 1350 | 1416 | 1444 | 1290 | 157 |
| 67 | 5 | 823 | 1036 | 1337 | 1424 | 1445 | 1292 | 164 |
| 67 | 6 | 803 | 1042 | 1336 | 1443 | 1457 | 1297 | 164 |
| 67 | 7 | 788 | 1057 | 1346 | 1467 | 1478 | 1317 | 175 |
| 67 | 8 | 784 | 1076 | 1368 | 1489 | 1501 | 1329 | 184 |
| 67 | 9 | 798 | 1096 | 1395 | 1505 | 1525 | 1346 | 189 |
| 67 | 10 | 822 | 1116 | 1422 | 1522 | 1548 | 1361 | 194 |
| 67 | 11 | 847 | 1134 | 1448 | 1538 | 1570 | 1381 | 198 |
| 67 | 12 | 870 | 1152 | 1469 | 1556 | 1592 | 1401 | 198 |
| 67 | 13 | 891 | 1169 | 1488 | 1577 | 1612 | 1421 | 201 |
| 66 | 14 | 910 | 1184 | 1505 | 1601 | 1631 | 1442 | 203 |
| 56 | 15 | 928 | 1199 | 1518 | 1624 | 1649 | 1446 | 217 |
| 52 | 16 | 947 | 1213 | 1530 | 1644 | 1665 | 1455 | 227 |
| 42 | 17 | 967 | 1229 | 1544 | 1658 | 1682 | 1441 | 245 |
| 41 | 18 | 989 | 1245 | 1562 | 1671 | 1699 | 1419 | 246 |

Table S8: Smoothed percentiles for 36 week gestation (N=70)

| N | days | 3^rd^ | 10^th^ | 50^th^ | 90^th^ | 97^th^ | mean | SD |
| --- | --- | --- | --- | --- | --- | --- | --- | --- |
| 70 | 1 | 1276 | 1328 | 1477 | 1482 | 1489 | 1438 | 77 |
| 70 | 2 | 1250 | 1300 | 1443 | 1454 | 1467 | 1405 | 74 |
| 70 | 3 | 1223 | 1275 | 1416 | 1437 | 1454 | 1380 | 73 |
| 70 | 4 | 1199 | 1256 | 1407 | 1435 | 1456 | 1372 | 81 |
| 70 | 5 | 1184 | 1243 | 1414 | 1446 | 1472 | 1375 | 95 |
| 70 | 6 | 1181 | 1239 | 1429 | 1466 | 1494 | 1382 | 107 |
| 70 | 7 | 1187 | 1241 | 1447 | 1491 | 1518 | 1389 | 118 |
| 70 | 8 | 1202 | 1253 | 1467 | 1517 | 1541 | 1407 | 123 |
| 70 | 9 | 1221 | 1274 | 1485 | 1541 | 1563 | 1416 | 123 |
| 70 | 10 | 1241 | 1299 | 1502 | 1562 | 1585 | 1433 | 124 |
| 70 | 11 | 1260 | 1323 | 1517 | 1582 | 1606 | 1447 | 127 |
| 70 | 12 | 1280 | 1345 | 1531 | 1599 | 1630 | 1455 | 125 |
| 70 | 13 | 1298 | 1364 | 1545 | 1616 | 1656 | 1462 | 133 |
| 70 | 14 | 1315 | 1379 | 1560 | 1634 | 1683 | 1479 | 145 |
| 58 | 15 | 1332 | 1392 | 1576 | 1653 | 1710 | 1459 | 156 |
| 45 | 16 | 1349 | 1405 | 1592 | 1675 | 1735 | 1454 | 171 |
| 34 | 17 | 1365 | 1420 | 1608 | 1698 | 1758 | 1460 | 188 |
| 25 | 18 | 1380 | 1435 | 1624 | 1721 | 1784 | 1481 | 187 |

**Postnatal growth percentile curves of preterm VLBW neonates with respect to their gestational age (29-36 weeks). Supplementary figures(F1-F8)**

Figure F1: Postnatal growth percentile curves for 29 week gestation preterm VLBW neonates admitted to the neonatal unit of Bahir Dar public hospitals, 2022 (N=48)

Figure F2: Postnatal growth percentile curves for 30 week gestation preterm VLBW neonates admitted to the neonatal unit of Bahir Dar public hospital, 2022  (N=31)

Figure F3: Postnatal growth percentile curves for 31 week gestation preterm VLBW neonates admitted to the neonatal unit of Bahir Dar public hospitals, 2022 (N=39)

 Figure F4: Postnatal growth percentile curves for 32 week gestation preterm VLBW neonates admitted to the neonatal unit of Bahir Dar public hospitals, 2022 (N=40)

 

Figure F5: Postnatal growth percentile curves for 33 week gestation preterm VLBW neonates admitted to the neonatal unit of Bahir Dar public hospitals, 2022 (N=62)

Figure F6: Postnatal growth percentile curves for 34 week gestation preterm VLBW neonates admitted to the neonatal unit of Bahir Dar public hospitals, 2022 (N=55)

Figure F7: Postnatal growth percentile curves for 35 week gestation preterm VLBW neonates admitted to the neonatal unit of Bahir Dar public hospitals, 2022 (N=67)

Figure F8: Postnatal growth percentile curves for 36 week gestation preterm VLBW neonates admitted to the neonatal unit of Bahir Dar public hospitals, 2022 (N=70)
