# Supplementary material for: Patterns of postnatal weight gain and its predictors among preterm very low birth weight neonates born in Bahir-Dar city public hospitals, 2022: A cross sectional study
Source: PLoS One. 2025 Feb 12;20(2):e0315573. doi: 10.1371/journal.pone.0315573 (PMC11819586; doi:10.1371/journal.pone.0315573)
Supplement: S1 Questionnaire — (DOCX) [file pone.0315573.s002.docx]

Questionnaires on patterns of postnatal weight gain in preterm very low birth weight infants born in Bahir-Dar public hospitals, 2022, cross sectional study

| MRN–––––––– | | | | |
| --- | --- | --- | --- | --- |
| Part 1: Socio-demographic questions | | | | |
| No | Question | | Possible answers | Skip pattern |
| 101 | Maternal age––––––– | |  |  |
| 104 | Number of children that the mother has | | 1 One  2 Two  3 >Three |  |
| 105 | Sex of the neonate | | 1 male  2 female |  |
| 106 | Birth weight (g)––––––––––––––– | |  |  |
| 107 | Minimum weight recorded | |  |  |
| 108 | Age at minimum weight recorded | |  |  |
| 109 | Percent of weight loss in average–––––– | |  |  |
| 110 | Age at regain Birth weight (day) –––––––– | |  |  |
| 111 | Average weight gain (GV) in g/kg/day––––– | |  |  |
| 112 | Discharge weight (g)––––––– | |  |  |
| 113 | net weight gain until discharge | |  |  |
| 114 | Z score at birth | | 1 < 1.29  2 >=1.29 |  |
| 115 | Z score at discharge | | 1 < 1.29  2 >=1.29 |  |
| Part 2: obstetrics characteristics | | | | |
| 201 | ANC visit by the mother | | 1. Yes 2. No |  |
| 202 | If yes for ANC, how many visit does she had | | 1. <= Two times 2. > Two times |  |
| 203 | Mode of delivery | | 1 C/S  2 SVD |  |
| 204 | Gestational age in wks.––––––––––––– | |  |  |
| 205 | Size for gestational age | | 1 AGA  2 SGA |  |
| Part 3: Variables related to comorbidities | | | | |
| 301 | Does the neonate diagnose with | RDS | 1 yes  2 no |  |
| 302 |  | Sepsis | 1 yes  2 no |  |
| 303 |  | NEC | 1 yes  2 no |  |
| 304 |  | Jaundice | 1 yes  2 no |  |
| 305 |  | Hypoglycemia | 1 yes  2 no |  |
| 306 |  | Anemia | 1 yes  2 no |  |
| 307 |  | Hypothermia | 1 yes  2 no |  |
| 308 |  | Others | 1 yes  2 no |  |
| 309 | duration of oxygen use in days–––––––– | | 1 less than 9 days  2 more than 9 days |  |
| 310 | Duration of hospital stay in days–––––––– | |  |  |
| 311 | Outcome of the neonate | | 1 death  2 discharge |  |
| Part 4: Feeding practice related questions | | | | |
| 401 | Type of milk fed on admission | | 1 Breast milk  2 Formula milk  3 Mixed |  |
| 402 | Initial volume of feeding in ml/kg––––– | | 1 2ml/kg  2 other |  |
| 403 | When to start the first feed | | 1 1st day  2 2^nd^ day and beyond |  |
| 404 | Initial method of feeding | | 1 Tube  2 Breastfeeding |  |
| 405 | Frequency of feeding | | 1 < every 3 hr  2 Every 3 hr  3 > every 3hr |  |
| 406 | When to reach full feed 150ml/kg | | 1 0–7  2 8–14  3 15–21  4 22+ |  |
| 407 | Does the neonate in KMC | | 1 yes  2 No |  |
