# Supplementary material for: Patterns of postnatal weight gain and its predictors among preterm very low birth weight neonates born in Bahir-Dar city public hospitals, 2022: A cross sectional study
Source: PLoS One. 2025 Feb 12;20(2):e0315573. doi: 10.1371/journal.pone.0315573 (PMC11819586; doi:10.1371/journal.pone.0315573)
Supplement: Attachment — Submitted filename: Ayehu, et al..docx [file pone.0315573.s003.docx]

Patterns of Postnatal Weight Gain and its Predictors among Preterm Very 2 Low Birth Weight Neonates Born in Bahir-Dar Public Hospitals Retrospective 3 Follow-up Study, 2022

Yihenew Ayehu, Dr. Solomon Hailemeskel, Jalane Mekonen

The study shows a clear direction. Data collection and analysis has been well explained. Results meet the aims. A few changes need to be considered.

Suggestions:

Line 22/23: Please explain ‘no feeding alternatives’ when tables show formula use.

Line 27: Infants were born from Apr 2020 to Apr 2022 (line 103-104), not 2022 alone.

Line 28, 99: Three types of retrospective studies accepted in literature are considered cross-sectional, case-control and cohort. This study would be better classified as a cohort study group rather than a retrospective ‘follow-up study’.

Line 29: Please avoid using nominal terms like ‘last’ two years and keep it specific to dates since the term ‘last’ will differ as per publication year.

Line 61: Please explain the methodology/ reasoning behind comparing post-natal weight gain with intrauterine growth curve.

Line 78-89: Nutritional requirement is defined by finite terms. Methods to meet that requirement can be different. The presentation needs to be clearer.

Line 175: Most of the results account for N= 412. Please explain where N = 206 was used for calculations.

Line 321: Discharge weight charting does not account for ‘long follow-up’

Please correct 2022 (N =412) to actual Apr 2021 to Apr 2022.

Multiple grammatical errors that need to go through proof reading/ an established proofreading software.

All references need to be re written according to specific style (APA or AMA)
