# Supplementary material for: Patterns of postnatal weight gain and its predictors among preterm very low birth weight neonates born in Bahir-Dar city public hospitals, 2022: A cross sectional study
Source: PLoS One. 2025 Feb 12;20(2):e0315573. doi: 10.1371/journal.pone.0315573 (PMC11819586; doi:10.1371/journal.pone.0315573)
Supplement: Attachment — Submitted filename: Authors response to reviewers comment.docx [file pone.0315573.s004.docx]

Yihenew Ayehu Dessie

Email: [filimonayehu21@gmail.com](mailto:filimonayehu21@gmail.com)

Dear Luisa Anna Denkel

We would like to thank you for the insightful comments and suggestions provided for our manuscript titled "[**patterns postnatal weight gain and its predictors among preterm very low birth weight neonates in Bahir Dar city public hospitals: A cross sectional study**]". We have carefully considered each point raised and have made the necessary revisions to address all concerns. Below is a detailed response to the comments.

**Editor Comment 1:** Please consult a statistician and revise the statistical analyses accordingly. The binomial regression analysis needs to be reported appropriately, especially the explanation on which category the aRR are referring to (APWG or PPWG, Table 5).  Further, definition of “adequate and poor weight gain” must be thoroughly explained as all risk factor analyses and conclusions are based on this definition. Please cite appropriate references.

**Response to Editor Comment 1:** Thank you for your insightful comments and suggestions on our manuscript. We have carefully considered your feedback and consulted with a statistician to address the concerns raised.

**Statistical Analysis:** You mentioned the need to revise the statistical analyses, particularly regarding the reporting of binomial regression analysis and clarification on the category to which the adjusted relative risks (aRR) refer (APWG or PPWG, Table 5). Upon consulting with a statistician, we revised our approach and conducted a binary logistic regression analysis instead of binomial regression. This method was deemed more appropriate for our dataset and research questions.

**Definition of "Adequate and Poor Weight Gain":** Regarding the definition of "adequate and poor weight gain," we appreciate your emphasis on the importance of clarity in this regard, as it underpins all risk factor analyses and conclusions in our study. To support our definition, we have cited relevant literature

**Definition Clarification:** We define "adequate postnatal weight gain" as achieving a rate of >=15 grams per kilogram per day (g/kg/day) among preterm very low birth weight neonates. This definition aligns with established literature on neonatal growth metrics (5,16,21).

**Editor Comment 2:** This is not a follow-up study. The manuscript and the title of the manuscript need to be revised accordingly.

**Response to Editor Comment 2:** Thank you for your constructive feedback regarding the study type and manuscript title. We appreciate your insights, which have prompted us to reevaluate and clarify these aspects. Upon careful consideration of your feedback, we recognize that our study design aligns more appropriately with a cross-sectional approach.

**Revised Title:** "Patterns of Postnatal Weight Gain and Predictors among Preterm Very Low Birth Weight Neonates: A Cross-Sectional Study"

**Editor Comment 3:** The recommendations to healthcare provider do not represent the study results.  “Warming” has not been included in the analyses but recommended as prevention measure for growth failures. The same applies to “Initiating trophic feeding on the first day of life”, timely treatment of comorbidities (except for RDS), hospital stays. Instead, frequency of feeding (<= every 3 hours) seems to be an effective measure for appropriate postnatal weight gain and should be recommended. This needs to be revised accordingly.

**Response to Editor Comment 3:** Thank you for your valuable feedback and for highlighting the discrepancies between the study results and the recommendations provided. We appreciate the opportunity to clarify and revise our manuscript based on your insights. **Emphasize on the importance of time to initiate the first feeding, birth z-score, mode of delivery, and effective management of RDS** as effective measures for promoting appropriate postnatal weight gain. These are highlighted as key recommendations in our revised manuscript.

**Editor Comment 4:** The structured questionnaire used must be included in the supplementary material.

**Response to Editor Comment4:** Thank you for your valuable feedback and for highlighting the need for additional materials to enhance the transparency and comprehensiveness of our study. We appreciate the opportunity to improve our manuscript based on your suggestions.

In response to your request, we have included the structured questionnaire used in our study in the supplementary material of the revised submission.

**General remarks:**

**Editor Comment 5:** Please use the abbreviations (VLBW) instead of the written out terms once they have been introduced.

**Response to Editor Comment 5:** Thank you for the feedback. "Very Low Birth Weight (VLBW)" is used consistently throughout the manuscript after it has been introduced.

**Editor Comment6:** Figure 1: Please revise Fig 1 (for spelling mistakes and skewed lines)

**Response to Editor Comment 6:** Thank you for pointing that out. We have removed Figure 1 and have described the sampling procedure in the text to clarify the information previously presented in the figure."

**Specific remarks**

**Editor Comment 7:** Line 49ff: This paragraph is not fully correct and needs revision. For VLBW definition, please check the bibliography, did you cite the correct references?

**Response to Editor Comment 7:** Thank you for your feedback. We have updated the paragraph to accurately define preterm very low birth weight (VLBW) by emphasizing both birth weight and gestational age.

**Editor Comment 8:** Abstract: The abbreviation SVD needs to be written out before its first use.

**Response to Editor Comment 8:** Thank you for your feedback. We have replaced the abbreviation "SVD" with "mode of delivery" in the abstract and introduced the term "SVD" as spontaneous vaginal delivery before its first use.

**Editor Comment 9:** Line 68: please correct “challenging” or “is a challenge”

**Response to Editor Comment 9:** Thank you for the comment. We have corrected the wording from “challenging” to “is a challenge”

**Editor Comment 10:** Line 70: Please introduce the term “NEC”.

**Response to Editor Comment 10:** Thank you for your feedback. We have introduced the term "NEC" as "necrotizing enterocolitis"

**Editor Comment 11:** Line 82: Please include a reference for this statement (“Although postnatal weight gain is a common problem in preterm very low birth weight infants in resource-limited countries”).

**Response to Editor Comment 11:** Thank you for your feedback. We have included a reference to support the statement, regarding postnatal weight gain being a common issue in preterm very low birth weight infants in resource-limited countries.

**Editor Comment 12:** Line 85: Please remove “in Ethiopia” and add another sentence to report that this study was conducted in a low resource country, namely Ethiopia”.

**Response to Editor Comment 12:** Thank you for your feedback. We have removed "in Ethiopia" and added a sentence indicating that the study was conducted in a low-resource country, specifically Ethiopia. Additionally, we have included a reference to support the statement regarding the high burden of postnatal weight gain in resource-limited countries.

**Editor Comment 13:** Line 88: Please add “in Bahir Dar public hospitals in Ethiopia”.

**Response to Editor Comment 13:** Thank you for your feedback. We have added "in Bahir Dar public hospitals in Ethiopia" to specify the study location.

**Editor Comment 14:** Line 95: Please cite a reference for the national guidelines of Ethiopia.

**Response to Editor Comment 14:** Thank you for your feedback. We have cited the reference for the national guidelines of Ethiopia.

**Editor Comment 15:** Line 96: Please introduce the abbreviation NG for NG tubes.

**Response to Editor Comment 15:** Thank you for your comment. We have introduced the abbreviation "NG" for nasogastric tubes.

**Editor Comment 16:** Line 99: This is NOT a follow-up study, but a retrospective cohort study. Please also remove the term “follow-up” from the title.

**Response to Editor Comment 16:** Thank you for your feedback and for pointing out the mischaracterization of our study design. We apologize for the oversight.

In response to your comments, we have revised our manuscript to correctly describe our study as a cross-sectional study rather than a retrospective cohort study. We have also removed the term "follow-up" from the title and any other relevant sections of the manuscript.

**Editor Comment 17:** Line 100: What does the study period (June 12 – July 20, 2022) refer to? Is this the time period when the retrospective study was conducted? Please report the time period when children were born a how long they have been followed up. Did you include all preterm infants that fulfilled the inclusion criteria?

**Response to Editor Comment 17:** Thank you for your detailed feedback and for seeking clarification on the study period and methodology.

The study period (June 12 – July 20, 2022) refers to the timeframe during which we conducted the data collection for our cross-sectional study. This period does not correspond to the birth dates of the children or the duration of their follow-up. To clarify, neonates admitted from April 2020 to April 2022 were included in our study. We selected the neonates using systematic random sampling with an interval of every 2 admissions, as detailed in the manuscript's sampling procedure section.

**Editor Comment 18:** Line 106: the phrase “were died or discharged” must be corrected.

**Response to Editor Comment 18:** Thank you for your detailed feedback and for pointing out the necessary correction.

In response to your comment, we have corrected the phrase “were died or discharged” to the appropriate phrasing. The revised sentence now reads “had died or were discharged.”

**Editor Comment 19:** Line 115: Please cite a reference for the two-point average model used.

**Response to Editor Comment 19:** Thank you for your detailed feedback and for highlighting the need to provide a reference for the two-point average model used in our study.

In response to your comment, we have added a citation for the two-point average model to the manuscript.

**Editor Comment 20:** Line 121: How many data collectors worked for the study? I understood that this retrospective analysis was based on chart review only, no measurements of weigth were conducted by the data collectors? Is this correct? What did the teaching of data collectors imply? Please specify this.

**Response to Editor Comment 20:** Thank you for your detailed feedback and for seeking clarification on the data collection process.

Data collection was carried out by four trained BSc nurses under the supervision of two MSc neonatal nurses. The study was a retrospective analysis based solely on chart reviews; no direct measurements of weight were conducted by the data collectors. The role of the data collectors was to review and record data from medical charts accurately. The training provided to the data collectors included instruction on how to systematically and accurately review the charts and record the relevant information needed for our study.

**Editor Comment 21:** Line 166: Please add a “dot” at the end of the sentence.

**Response to Editor Comment 21:** Thank you for your feedback. We have added a period at the end of the sentence. We appreciate your careful review and the opportunity to make this correction.

**Editor Comment 22:** Line 175: Please explain why only 206 VLBW were included in the analyses (while 412 were selected and recruited). Please include the exclusion steps /criteria to Fig 1.

**Response to Editor Comment 22:** Initially, we calculated the sample size as 423. After excluding 11 charts with incomplete data, the number was reduced to 412. The 206 VLBW neonates mentioned in the manuscript is typing error.

**Editor Comment 23:** Line 176: What do the authors mean by “response rate”?

**Response to Editor Comment 23:** Thank you for your feedback and for seeking clarification regarding the term “response rate.”

In our study, the “response rate” refers to the proportion of eligible preterm VLBW neonates who were included in the final analysis out of a total sample.

**Editor Comment24:** Line 177: Do the authors “Caesarian section” or “Caesarian” by “Section” Please revise in the entire manuscript.

**Response to Editor Comment 24:** Thank you for your feedback. We have reviewed the manuscript and noted the need to clarify and standardize the terminology.

We revised the entire manuscript to consistently use the term "Caesarean section" instead of “Caesarian section” or “Caesarian.” These changes are applied throughout the document to ensure clarity and consistency.

**Editor Comment 25:** Line 178: Please introduce the abbreviation “ANC”.

**Response to Editor Comment 25:** Thank you for your feedback. We acknowledge the need to introduce and clarify the abbreviation “ANC.”

We revised the manuscript to introduce “ANC” as an abbreviation for “Antenatal Care” at its first occurrence. This will ensure that readers clearly understand the term throughout the document.

**Editor Comment 26:** Line 191: Please revise spelling of “There”.

**Response to Editor Comment26:** We have revised the spelling of "There."

**Editor Comment 27:** Table 1, Table 2, Table 4: Please include p-values for comparison of adequate and poor weight gain.

**Response to Editor Comment 27:** Thank you for your feedback regarding Tables 1, 2, and 4.

In response to your comment, we updated Tables 1, 2, and 4 to include p-values for the comparison of adequate and poor weight gain. This will provide a clearer understanding of the statistical significance of the differences observed between the two groups.

**Editor Comment 28:** Please explain and name all references you used for your definition of “adequate and poor weight gain”. This is crucial to report as your risk factor analyses is based on this definition.

**Response to Editor Comment 28:** Thank you for your feedback and for highlighting the importance of clearly defining “adequate and poor weight gain.”

In response to your comment, we revised the manuscript to include citations for the references used in defining “adequate and poor weight gain.”

**Editor Comment 29:** Line 223: This part belongs to the method’s section. Please revise this accordingly.

**Editor Comment 29:** Thank you for your feedback.

In response to your comment, we have revised the manuscript to move the content to the methods section.

**Editor Comment 30:** Line 263: Please check the reported average postnatal weight gain of “11.43.8 g/kg/d”.

**Response to Editor Comment 30:** Thank you for your feedback

In response to your comment, we have corrected the reported average postnatal weight gain from “11.43.8 g/kg/d” to the correct value of “11.1 g/kg/d.” We updated the manuscript accordingly.

**Editor Comment 31:** Table 5:This table is hard to read / interpret. It is not clear, for which category the authors report CRR / aRR (APWG or PPWG). Please report postnatal weight gain by the following categories: appropriate weight gain (= 1), while poor weight gain (= no appropriate weight gain, referenced as “0”) or mark in the table which category represents the reference.

**Response to Editor Comment 31:** Thank you for your insightful feedback on Table 5. We value your thorough review and the opportunity to improve our manuscript.

Code: "Appropriate weight gain" as “1” and "Poor weight gain" as “0.”

We focus on adequate weight gain in our analysis because preterm very low birth weight neonates commonly struggle with poor postnatal weight gain. Our goal is to identify the factors that contribute to achieving adequate weight gain.

**Editor Comment 32:** RDS: Using “Yes” as reference is difficult to interpret for the reader.

**Response to Editor Comment 32:** Thank you for your feedback regarding the use of “Yes” as the reference category for RDS (Respiratory Distress Syndrome).

We acknowledge that using “Yes” as a reference may be challenging for readers to interpret. To enhance clarity, we revised the manuscript to use “No RDS” as reference category.

**Editor Comment 33:** Line 342: “Warming” has not been included in the analyses. How can the authors recommend this as prevention measure for growth failures? The same applies for “Initiating trophic feeding on the first day of life”, timely treatment of comorbidities (except for RDS), hospital stays. Instead, frequency of feeding (<= every 3 hours) seem to be an effective measure for appropriate postnatal weight gain.

**Response to Editor Comment 33:** Thank you for your feedback and for highlighting the need to align our recommendations with the analyses conducted.

In response, we have revised our recommendations to reflect the findings from our analyses. Specifically, we now recommend measures related to vaginal delivery, birth z-score, early initiation of the first feed, and timely treatment of RDS as an effective measure for promoting appropriate postnatal weight gain.

**Dear: Reviewers**

We would like to thank you for the insightful comments and suggestions provided for our manuscript titled "[**patterns postnatal weight gain and its predictors among preterm very low birth weight neonates in Bahir Dar city public hospitals: A cross sectional study**]". We have carefully considered each point raised and have made the necessary revisions to address all concerns. Below is a detailed response to the comments.

**Reviewer 1 comment 1:** Background: Line 49: Paraphrase it….. In such way…..”Very low birth weight (VLBW) preterm infant is defined by the World Health Organization 50 (WHO) as those infants born weighing less than 1500 grams before 37 completed weeks of gestation.”

**Authors’ response:** Thank you for your feedback. We have paraphrased the sentence as suggested. We appreciate your guidance in improving the clarity of our manuscript.

**Reviewer 1 comment 2:** Line 63: Flow of ideas are not coherent, such as similarly….. What does it links from the previous paragraph

**Authors’ response:** Thank you for your feedback regarding the coherence and flow of ideas in the manuscript. We have reviewed and revised the text to improve the logical flow and connectivity between paragraphs. We appreciate your constructive feedback and the opportunity to refine our manuscript.

**Reviewer 1 comment 2:** Line 70: Some previously defined abbreviations/ Acronyms such as NEC.

**Authors’ response:** Thank you for pointing out the need to define previously mentioned abbreviations. We have defined the term “NEC” (Necrotizing Enterocolitis) in the revised manuscript.

Generally, the background section is well written on the view of known scientific guidelines and books. However, the disparities and previous findings of literatures was not explored. Due to this, why the research was done is not clear. It is my suggestion to revise the background section by incorporating strong statement of the problem and justifications with its implication on further to the scientific community.

Thank you for your insightful feedback on the background section of our manuscript. We appreciate your suggestion to incorporate a stronger statement of the problem and provide a clearer justification for our research.

In response, we have revised the background section to better address the disparities and previous findings in the literature. We have included a more detailed discussion on the existing gaps and the significance of our research, highlighting its implications for the scientific community.

**Reviewer 1 comment 3:** Methods,

Design: why it is follow up study? Why not Cohort? I am happy to know their difference.
The time period for the follow up study was nearly month (Even though the study incorporates 2 years charts). Could be a follow up study with this period? It seems cross-sectional chart review than follow up study? Nothing was followed by this study. How the study assured that the data were collected before the outcome occurred? For example, comorbid condition may occurred after the exacerbation of the weight loss.

**Authors’ response:** Thank you for your constructive feedback regarding the study design. Upon careful consideration and consultation with a statistician, we have determined that our study aligns more appropriately with a cross-sectional design rather than a follow-up or cohort study.We have revised the manuscript accordingly to accurately reflect this design change and ensure consistency throughout the document.

**Reviewer 1 comments 4:** Sample size calculation?????? And Sampling techniques????

**Authors’ response:** Thank you for your feedback regarding sample size calculation and sampling techniques.

In response, we have included a detailed description of the sample size calculation in the revised manuscript, outlining the methodology used to determine the appropriate number of participants. Additionally, we have provided a comprehensive explanation of the sampling techniques employed in the study to ensure clarity on how participants were selected.

**Reviewer 1 comments 5:** Line 113: We are not sure whether daily weight was recorded.

**Authors’ response:** Thank you for your feedback. We have neonatal intensive care guidelines in Ethiopia that recommend recording daily weights.

**Reviewer 1 comments 6:** Line 117/124: Paraphrase and punctuate appropriately.

**Authors’ response:** Thank you for your feedback. We have paraphrased and corrected the punctuation in the revised manuscript.

**Reviewer 1 comments 7:** Line 129: Why you operationalized some terms such as Growth velocity, Growth failure; non used words in the study results.

**Authors’ response:** Thank you for your feedback. We appreciate your observation regarding the operationalized terms. In our study, "growth velocity" is used similarly to "postnatal weight gain from birth to discharge," which aligns with our dependent variable. Similarly, "growth failure" is associated with the discharge z-score.

**Reviewer 1 comments 8:** Line 144/147: It is not clear why such percentages were calculated, better to explain its advantages for the study findings.

**Authors’ response:** Thank you for your feedback regarding the calculation of percentages. To clarify, we used the 3rd, 5th, 10th, 50th, and 97th percentiles to plot postnatal growth curves, as illustrated in the supplementary graphs. The calculation of these specific percentiles helps us to clearly visualize and compare the growth patterns of preterm VLBW neonates.

**Reviewer 1 comments 9:** Line 154: Why VIF used? To check confounding????

**Authors’ response:** Thank you for your question regarding the use of the Variance Inflation Factor (VIF). We used VIF to assess multicollinearity among the predictor variables in our analysis. Specifically, VIF helps to identify whether any predictors are highly correlated with each other, which can affect the reliability of the regression coefficients and potentially obscure the effects of individual variables.

**Reviewer 1 comments 10:** Line 171: The data reliability should be checked. I don’t think such number of twin/triple, death or discharged before 7 days among VLBW neonates in your study set up.

**Authors’ response:** Thank you for your feedback regarding data reliability and the high numbers of twins, triplets, deaths, and early discharges among VLBW neonates in our study. We acknowledge the importance of verifying data accuracy. Being a twin or triplet is a known risk factor for preterm very low birth weight (VLBW) infants, and a significant proportion of these infants do not survive their first week of life in low- and middle-income countries (LMICs).

**Reviewer 1 comments 11:** Line 175: What are the actual respondents, make clear. Is 199 or 206 or 412 or Etc.

**Authors’ response:** Thank you for your comment regarding the number of actual respondents. We have revised the manuscript to clearly indicate that 412 is the correct number of preterm VLBW neonates included in the study. The discrepancies were due to a typing error.

**Reviewer 1 comments 12:** Line 177/178: Sections??? Most neonates of neonates????

**Authors’ response:** Thank you for pointing out the issue with the phrasing. We have revised the manuscript to correct the text and have used "Most neonates" to ensure clarity.

**Reviewer 1 comments 13:** Line 187: Initially your study excluded 206 participants, but all your write up included all 412 charts. Please write again and make consistent writing throughout the documents.

**Authors’ response:** Thank you for highlighting the inconsistency regarding the number of participants. To address this, we have revised the manuscript to ensure consistency throughout. We now clearly state that the final analysis included all 412 charts, and we have updated the text to reflect this accurately.

**Reviewer 1 comments 14:** Table 2: Is determining the time to reach full volume feeds the study’s objective? This may need another type of modeling and analysis.

**Authors’ response:** We appreciate the comment. The inclusion of the average time to reach maximum feeding volume in Table 2 aligns with our study's focus on factors influencing neonatal weight gain. This metric is crucial for managing feeding schedules, ensuring timely nutrient intake, and assessing feeding tolerance and gastrointestinal maturity. These factors are essential for optimizing growth outcomes and guiding clinical decisions on feeding protocols.

Line 213: Did you think the comorbidity precedes the weight loss or the reverse?

**Authors’ response:** Thank you for your valuable comment. Physiological weight loss in the first week of life is common and occurs in all neonates, primarily due to the loss of excess extracellular fluid and the transition to extrauterine life. However, comorbidities such as infections, metabolic disorders, or respiratory issues can exacerbate this weight loss. These conditions impair the neonate's ability to intake and retain adequate nutrition, leading to greater weight loss compared to healthy neonates.

**Reviewer 1 comments 15:** Table 4: Is the objective of your study?

**Authors’ response:** Thank you for your comment. The objective of our study is to examine factors affecting postnatal weight gain in preterm VLBW neonates. Feeding practice is a crucial factor influencing weight gain. Different feeding methods (breastfeeding, formula feeding, mixed feeding) directly impact nutritional intake and growth outcomes. Presenting this data is essential to understanding and improving postnatal weight gain in this population.

**Reviewer 1 comments 16:** Line 223-234: Did the variables full filled the model assumption? Log-binomial model assumptions???? And model adequacy???

**Authors’ response:** Thank you for your comment. Based on the statistician's recommendation, we switched to binary logistic regression. We assessed the model fitness using the Hosmer-Lemeshow test, which confirmed that the model assumptions were met and the model was adequate.

**Reviewer 1 comments 17:** Line 259: Still the study discussed opposite to the initial objectives of the study. And please calculate the confidence interval for poor and adequate proportion of weight gain Preterm VLBW neonates.

**Authors’ response:** Thank you for your feedback. We have revised the discussion to align more closely with the initial objectives of the study. Additionally, we have calculated and included the confidence intervals for both poor and adequate proportions of weight gain in preterm VLBW neonates.

**Reviewer 1 comments 18:** Generally, the discussion section was shallow and use inappropriate discussion techniques, for example, if the population was different, discussing a different population is inappropriate.

**Authors’ response:** Thank you for your feedback. We have revised the discussion section to address its depth and relevance. We have removed any inappropriate references to different populations and focused the discussion on our study’s specific population and findings. These changes enhance the clarity and applicability of our analysis.

**Reviewer 1 comments 19:** References: Most references used were outdated, please updated with the past five years.

**Authors’ response:** Due to limited new research on postnatal weight gain in preterm VLBW neonates over the past five years, some older references remain. We have included the most relevant and recent studies to reflect current knowledge.

**Comments of reviewer #2:** While none of the results presented are surprising, it is important for country specific data regarding this important issue be available. My only concern is lumping together late and early preterm infants given that these are very different in their comorbidities and ability to tolerate enteral feeding.

**Authors’ response:** Thank you for your comment. We understand the importance of differentiating between early and late preterm infants due to their distinct comorbidities and feeding tolerances. However, we grouped them together because we consider gestational age a significant predictor of postnatal weight gain. By including gestational age in our analysis, we can account for its impact on growth outcomes across the preterm spectrum, providing valuable insights applicable to a broad clinical context. This approach enhances our understanding of general trends and predictors of weight gain in preterm VLBW infants in Ethiopia.

**Authors’ response:** Thank you for your positive feedback on the study aims and methodology. We appreciate your suggestions for improving the writing, presentation of data, and references. We revised the manuscript accordingly to enhance clarity and ensure that the references are formatted correctly. We carefully reviewed the detailed comments in the attachment and make the necessary improvements to meet the journal's standards.

**Comments on the attached file**

**Reviewer Comment 1:** Line 22/23: Please explain ‘no feeding alternatives’ when tables show formula use.

**Author’s response1:** Thank you for your comment. In low and middle-income countries like Ethiopia, “no feeding alternatives” refers to the limited access to specialized feeding options for very low birth weight (VLBW) infants. While formula feeding is utilized, it often lacks the specific nutrients required for VLBW infants due to the unavailability of specialized preterm formulas. Additionally, challenges such as inadequate infrastructure for formula preparation, limited follow-up and monitoring, and economic constraints further impact the effectiveness of formula feeding. These factors highlight the broader issue of insufficient feeding alternatives and support systems for VLBW infants in these settings.

**Reviewer Comment 2:** Line 27: Infants were born from Apr 2020 to Apr 2022 (line 103-104), not 2022 alone.

**Author’s response 2:** Thank you for your detailed feedback and for seeking clarification on the study period and methodology. The study period (June 12 – July 20, 2022) refers to the timeframe during which we conducted the data collection for our cross-sectional study. This period does not correspond to the birth dates of the children or the duration of their follow-up. To clarify, neonates admitted from April 2020 to April 2022 were included in our study. Apologies for the typing error and thank you for helping us correct it.

**Reviewer Comment 3:** Line 28, 99: Three types of retrospective studies accepted in literature are considered cross-sectional, case-control and cohort. This study would be better classified as a cohort study group rather than a retrospective ‘follow-up study’.

**Author’s response 3:** Thank you for your feedback. Upon careful consideration, we recognized that our study design aligns more appropriately with a cross-sectional approach.

**Reviewer Comment 4:** Line 29: Please avoid using nominal terms like ‘last’ two years and keep it specific to dates since the term ‘last’ will differ as per publication year.

**Author’s response 4:** Thank you for your suggestion. We have revised the text to avoid using nominal terms.

**Reviewer Comment 5:** Line 61: Please explain the methodology/ reasoning behind comparing post-natal weight gain with intrauterine growth curve.

**Author’s response 5:** Thank you for your comment. Comparing post-natal weight gain with the intrauterine growth curve is important for evaluating how well neonates are growing after birth. The intrauterine growth curve provides a reference for expected fetal growth, which helps us assess whether post-natal weight gain is on track. This comparison allows us to identify any deviations from expected growth patterns, such as inadequate or excessive weight gain, and ensures that neonates are receiving appropriate care and interventions if needed.

**Reviewer Comment 6:** Line 78-89: Nutritional requirement is defined by finite terms. Methods to meet that requirement can be different. The presentation needs to be clearer.

**Author’s response 5:** Thank you for your insightful feedback. We have revised the paragraph for clarity and comprehensibility.

The nutritional requirements for preterm very low birth weight (VLBW) infants to achieve adequate weight gain are significantly influenced by the type, amount, and frequency of feeding during their initial hospitalization. These nutritional practices, often guided by expert opinions, aim to meet growth standards that might not always be optimal. Various factors, such as the volume of feeding, the type of feeding (whether breast milk or formula), and the feeding frequency, affect these requirements. Therefore, this study is important as it addresses the gap in evidence on postnatal weight gain patterns and predictors in preterm VLBW infants, particularly in Ethiopia, where research on this topic is limited.

**Reviewer Comment 7:** Line 175: Most of the results account for N= 412. Please explain where N = 206 was used for calculations.

**Author’s response 7:** Thank you for your comment. The number 206 mentioned in the manuscript is a typing error. The correct N is 412, which we used for all results. We initially calculated 423 we calculated the sample size as 423. After excluding 11 charts with incomplete data, the number was reduced to 412. We have corrected this error in the manuscript.

**Reviewer Comment 8:** Line 321: Discharge weight charting does not account for ‘long follow-up’

**Author’s response 8:** Thank you for your comment. The weight data collected up to discharge is indeed valuable for assessing immediate growth patterns and outcomes in preterm VLBW infants. As strength of our study, we highlight that the comprehensive monitoring of neonatal growth from admission through to discharge allows us to capture detailed and relevant weight data during this critical period of development. This approach provides important insights into the effectiveness of early interventions and nutritional practices.

**Reviewer Comment 9:** Please correct 2022 (N =412) to actual Apr 2021 to Apr 2022.

Author response: Thank you for pointing this out. The study period (June 12 – July 20, 2022) To clarify, neonates admitted from April 2020 to April 2022 were included in our study. The reference to 2022 was intended to denote the study year.

**Reviewer Comment 10:** Multiple grammatical errors that need to go through proof reading/ established proofreading software.

**Author’s response 10:** Thank you for highlighting this issue. We have addressed the grammatical errors in the manuscript by using established proofreading software. We have carefully reviewed and revised the manuscript to improve its overall quality and readability.

**Reviewer Comment 11:** All references need to be re written according to specific style (APA or AMA)

**Author’s response 11:** Thank you for your comment. We used the Vancouver style for the references as it adheres to the journal's guidelines. If the journal requires APA or AMA style, we updated the references accordingly.
