# Supplementary material for: Patterns of postnatal weight gain and its predictors among preterm very low birth weight neonates born in Bahir-Dar city public hospitals, 2022: A cross sectional study
Source: PLoS One. 2025 Feb 12;20(2):e0315573. doi: 10.1371/journal.pone.0315573 (PMC11819586; doi:10.1371/journal.pone.0315573)
Supplement: Attachment — Submitted filename: Response to Reviewers.docx [file pone.0315573.s005.docx]

Yihenew Ayehu Dessie

Email: [filimonayehu21@gmail.com](mailto:filimonayehu21@gmail.com)

Dear: Reviewer's

Thank you for your insightful comments and for the continued review of our manuscript titled "Patterns of Postnatal Weight Gain and Its Predictors Among Preterm Very Low Birth Weight Neonates in Bahir Dar City Public Hospitals: A Cross-Sectional Study." Each point raised has been carefully considered, and we have made the necessary revisions accordingly.

**Motivation for the research:** during our clinical attachments, we observed that daily weights of neonates were measured and recorded in vital sign sheets according to neonatal guidelines. However, there was no systematic plotting of whether these infants experienced significant weight loss or adequate weight gain. This lack of visualization and analysis of weight trends sparked our interest in understanding the patterns of postnatal weight gain among very low birth weight (VLBW) preterm infants. Therefore, this study aims to assess these weight gain patterns and identify predictors among preterm VLBW neonates, ultimately contributing to improved management practices in neonatal care. (See supplementary files). Below is a detailed response to the comments.

**Comment 1:**  Still, I have concerns about the general idea flow, and the reason for the study being done was not well elaborated. Methodologically, the reliability and accuracy of the study's data are questionable.

**Author response:** Thank you for your insightful feedback. We have made the following adjustments to improve the clarity and rationale of the study:

We have revised our background to clarify the rationale by emphasizing the high burden of preterm VLBW neonates in Ethiopia and the gaps in current research, such as the lack of population-specific growth data. These additions underline the importance of the study and its relevance. We also reviewed our methodology to improve clarity, aiming to enhance the perceived reliability and accuracy of our data by highlighting the rigor in data collection. Regarding the daily measure of weights, it is standard practice in neonatal care unit in our setting. Thank you for this valuable feedback.

**Reviewer #3: comment 1**: Please define “early” vs “late” preterm deliveries.

**Author response:** Thank you for your comment. We have added the definitions of "early preterm" and "late preterm" to the manuscript for clarity. Specifically, we define **early preterm** as infants born 34 **completed weeks of gestation,** and **late preterm** as those born between **34 and 36 completed weeks of gestation.** This addition has been made in the in the operational definitions section of the manuscript.

**Comment 2:**  The “number of children that the mother has”, although relevant, does not add up to n= 412. The information does not appear to contribute to final data or discussion and may be dropped, if incomplete.

**Response to Comment 2:** Thank you for your careful review. The discrepancy in the “number of children that the mother has” was caused by the unintentional deletion of some cells while removing track changes for submission. We have now restored the missing data. Additionally, we have incorporated the number of antenatal care (ANC) visits and the presence of ANC visits in the table to further enhance the relevance of the maternal characteristics in our analysis. The revised table and manuscript are now updated accordingly.

**Comment 3:**  Although it appears to be weight loss within the first week of life, please define “average weight loss" in table 3

**Response to Comment 3:** Thank you for your valuable feedback. We have included a clear definition of "average weight loss" in the operational definitions section of the manuscript. It is now defined as the total weight lost from birth until the neonate regains birth weight, divided by the number of days it took to regain that weight. This provides a daily average of weight loss during that period. We appreciate your suggestion, which has helped enhance the clarity of our manuscript.

**Comment 4:**  Data for Z score at birth is flipped in table 3

**Response to Comment 4:** Thank you for pointing out this issue. Upon review, we realized that the Z-score data at birth in Table 3 was indeed incorrectly presented. We have corrected the table by flipping the data, and it now accurately reflects the Z-scores as intended. The revised Table 3 has been updated in the manuscript for your review. We appreciate your attention to detail and apologize for any confusion this may have caused.

**Comment 5:** Please recheck p=value calculation for discharge weight category in table 3.

**Response to Comment 5:** Thank you for your valuable feedback regarding Table 3. Upon review, we believe the comment was directed towards the p-value calculation for the discharge z-score rather than the discharge weight category. After rechecking the statistical analysis, we identified a minor discrepancy in the p-value for the discharge z-score. The correct p-value has been recalculated and updated in the manuscript. This change has been highlighted in the revised version of Table 3

**Comment 6:** Please follow APA format for table titles

**Response to Comment 6:** Thank you for your feedback on the table titles.

We revised all the table titles in the manuscript to follow APA format. The table numbers are bolded, and the titles are now italicized and written in title case, as per APA guidelines. These changes have been implemented throughout the manuscript, and we have ensured consistency in formatting.

**Response to Comment 7:** Spelling and grammar re-checks needed. The letter “c” in word “city” should not be capitalized, for example. “Loss” instead of “lose” in table 3. Some other lines like 164-165 (birth weights don’t change; only weights do). Line 45, 46, 47; 134, etc.

Thank you for your valuable feedback regarding spelling and grammar. We have carefully reviewed and corrected the noted issues, including revising “City” to lowercase in titles and using “loss” rather than “lose” in Table 3. We also clarified that the term "loss" accurately reflects the initial, temporary decrease in neonates’ weight, which they later regain, as per the study’s focus on tracking weight trends. We have made further revisions on lines 45, 46, 47, and 134, as suggested.
