# Supplementary material for: Patterns of postnatal weight gain and its predictors among preterm very low birth weight neonates born in Bahir-Dar city public hospitals, 2022: A cross sectional study
Source: PLoS One. 2025 Feb 12;20(2):e0315573. doi: 10.1371/journal.pone.0315573 (PMC11819586; doi:10.1371/journal.pone.0315573)
Supplement: Attachment — Submitted filename: Response to Reviewers.docx [file pone.0315573.s006.docx]

Yihenew Ayehu Dessie

Email: [filimonayehu21@gmail.com](mailto:filimonayehu21@gmail.com)

Dear: Reviewer's

Thank you for your insightful comments and for the continued review of our manuscript titled "Patterns of Postnatal Weight Gain and Its Predictors among Preterm Very Low Birth Weight Neonates in Bahir Dar City Public Hospitals: A Cross-Sectional Study." Each point raised has been carefully considered, and we have made the necessary revisions accordingly.

**Comment 1:** Line 29: Please specify which mode of delivery (compared to what) and which z-scores were identified as “risk factors” (predictors) for postnatal weight gain. Please avoid colon (:) in the sentence.

**Authors’ response:** Thank you for your valuable feedback. We have revised the manuscript to specify the comparison in the mode of delivery, indicating that spontaneous vaginal delivery was compared to cesarean delivery. Additionally, we clarified the birth Z-score cutoff, specifying a Z-score greater than -1.29 as a predictor for postnatal weight gain. We also removed the colon as recommended.

**Comment 2:** Please do not use the term “babies” but “infants” throughout the entire manuscript

**Authors’ response:** Thank you for this guidance. We have reviewed the manuscript and replaced all instances of “babies” with “infants” to ensure consistency in terminology throughout the text.

**Comment 3:** Line 72: VLBW has already been introduced in line 41, please use the abbreviation after the first introduction.

**Authors’ response:** Thank you for pointing this out. We have reviewed the manuscript and ensured that "VLBW" is used consistently as an abbreviation after its first introduction.

**Comment 4:** Table 1: for gram, please use the abbreviation “g” instead of “gm”; Please re-check the spelling of the legend (small / large characters)

**Authors’ response:** Thank you for the detailed feedback. We have updated Table 1 to use the standard abbreviation “g” for gram instead of “gm.” Additionally, we have reviewed and corrected the spelling and capitalization in the table legend to ensure consistency with the recommended format.

**Comment 5:** Please re-check the manuscript for spelling mistakes, such as line 270 “Furthermore, Environmental factors […]”.

**Authors’ response:** Thank you for your careful review. We have thoroughly checked the manuscript for spelling and capitalization errors, including correcting "Environmental" to lowercase on line 270 and ensuring consistent formatting throughout the text.
